# Exogenous Nitric Oxide Reinforces Photosynthetic Efficiency, Osmolyte, Mineral Uptake, Antioxidant, Expression of Stress-Responsive Genes and Ameliorates the Effects of Salinity Stress in Wheat

**DOI:** 10.3390/plants10081693

**Published:** 2021-08-18

**Authors:** Ghalia S. H. Alnusairi, Yasser S. A. Mazrou, Sameer H. Qari, Amr A. Elkelish, Mona H. Soliman, Mohamed Eweis, Khaled Abdelaal, Gomaa Abd El-Samad, Mohamed F. M. Ibrahim, Nihal ElNahhas

**Affiliations:** 1 Department of Biology, College of Science, Jouf University, Sakaka 72388, Saudi Arabia; gshalnusairi@ju.edu.sa; 2 Business Administration Department, Community College, King Khalid University, Guraiger, Abha 62529, Saudi Arabia; ymazrou@kku.edu.sa; 3 Faculty of Agriculture, Tanta University, Tanta 31512, Egypt; 4 Biology Department, Al-Jumum University College, Umm Al-Qura University, Mecca 21955, Saudi Arabia; shqari@uqu.edu.sa; 5 Botany Department, Faculty of Science, Suez Canal University Ismailia, Ismailia 41522, Egypt; amr.elkelish@science.suez.edu.eg; 6 Botany and Microbiology Department, Faculty of Science, Cairo University, Giza 12613, Egypt; amradel807080@googelmail.com; 7 Plant Pathology and Biotechnology Laboratory, Excellence Center (EPCRS), Faculty of Agriculture, Kafrelsheikh University, Kafr Elsheikh 33516, Egypt; khaled.elhaies@gmail.com; 8 Department of Agronomy, Faculty of Agriculture, Ain Shams University, Cairo 11566, Egypt; Gomaa_abdelsamad@agr.asu.edu.eg; 9 Department of Agricultural Botany, Faculty of Agriculture, Ain Shams University, Cairo 11566, Egypt; Ibrahim_mfm@agr.asu.edu.eg; 10 Department of Botany and Microbiology, Faculty of Science, Alexandria University, Alexandria 21526, Egypt; nihal.elnahhas@alexu.edu.eg

**Keywords:** nitric oxide, salinity stress, antioxidant system, osmolytes, photosystem II, Na^+^/H^+^ antiporters, *Triticum aestivum* L.

## Abstract

Salinity stress is one of the major environmental constraints responsible for a reduction in agricultural productivity. This study investigated the effect of exogenously applied nitric oxide (NO) (50 μM and 100 μM) in protecting wheat plants from NaCl-induced oxidative damage by modulating protective mechanisms, including osmolyte accumulation and the antioxidant system. Exogenously sourced NO proved effective in ameliorating the deleterious effects of salinity on the growth parameters studied. NO was beneficial in improving the photosynthetic efficiency, stomatal conductance, and chlorophyll content in normal and NaCl-treated wheat plants. Moreover, NO-treated plants maintained a greater accumulation of proline and soluble sugars, leading to higher relative water content maintenance. Exogenous-sourced NO at both concentrations up-regulated the antioxidant system for averting the NaCl-mediated oxidative damage on membranes. The activity of antioxidant enzymes increased the protection of membrane structural and functional integrity and photosynthetic efficiency. NO application imparted a marked effect on uptake of key mineral elements such as nitrogen (N), potassium (K), and calcium (Ca) with a concomitant reduction in the deleterious ions such as Na^+^. Greater K and reduced Na uptake in NO-treated plants lead to a considerable decline in the Na/K ratio. Enhancing of salt tolerance by NO was concomitant with an obvious down-regulation in the relative expression of SOS1, NHX1, AQP, and OSM-34, while D2-protein was up-regulated.

## 1. Introduction

Being sessile, plants are frequently confronted with various environmental stresses, resulting in considerable alternation in metabolism, which leads to a serious threat within the yield and crop production [1,2]. Among the key stress factors, salinity seriously affects the growth and development of plants [3,4,5]. Nowadays, at global levels, the problem of salinity, particularly in the arid and semiarid regions, is increasing, and the problem has been intensified due to the continuous usage of salt-rich water for irrigation purposes [6,7]. Such agricultural malpractices have to lead to the continuous addition of excess toxic salts to soil, rendering the productive lands a saline wasteland. It has been estimated that 5–7% of the global land and approximately 20% of irrigated land areas are affected by high salinity [8,9,10]. Salinity stress proved to be detrimental to the growth and development of existing crops through induction of aberrations in physiological and biochemical processes, including chlorophyll synthesis, photosynthesis, respiration, and ion homeostasis [11,12,13]. Moreover, salinity stress negatively affects the metabolism, particularly the nitrogen or carbon assimilatory pathway, which in turn reflects reduced growth and yield [14,15,16].

It has been reported that the availability of excess toxic salts at the subcellular level triggers the excessive generation of reactive oxygen species (ROS), resulting in the induction of oxidative stress [1,17,18]. These ROS include radicals such as superoxide, hydrogen peroxide, hydroxyl, and peroxide, which are injurious to plant metabolism and growth at higher concentrations [19,20,21]. The accumulation of ROS in plant tissue can result in the oxidation of lipids [22,23], proteins [20,24], nucleic acids [25,26], and chlorophyll [27,28], thereby disturbing the structural and functional integrity of cells. To avoid the deleterious impact of accumulated ROS and also prevent their further generation, several defensive mechanisms are being evolved to improve cellular functioning [29,30].

These mechanisms include the selective absorption of mineral ions for osmotic adjustment and up-regulation of antioxidant and osmoprotective agents accumulated in the cytoplasm and organelles [11,31]. Additionally, osmolytes such as proline, glycine betaine, and sugars accumulate to maintain cellular functioning by making the cells osmotically stable. This takes place through the maintenance of the cell water content and assists in scavenging ROS, hence maintaining the enzyme structure and functioning [32,33]. Furthermore, the antioxidant defense system contributes to the neutralization of toxic ROS for preventing oxidative damage effects [19,34].

Nitric oxide (NO) is an essential radical molecule implicated in several physiological and biochemical functions of plants [13,35]. NO is involved in growth and development, as well as in defense responses to a variety of abiotic stresses, including salinity [36,37]. Working with different plant species, researchers have considered NO as an endogenous signaling molecule implicated in the regulation and coordination of the signaling network [38,39]. NO is the leading molecule for several physiological and adaptive biochemical changes [40]. The resultant effects, whether beneficial or deleterious, have been shown depending on the concentration and the site of production [41,42].

Wheat (*Triticum aestivum* L.) is one of the most important and strategic cereal crops in temperate zones. It is one of the preferred meals that are being used by approximately 36% of the whole population. Worldwide, wheat supplies almost 55% of the carbohydrates and 20% of the food calories consumed globally [43]. During the last 20 centuries, the demand for wheat has doubled due to urbanization and industrialization century. Salinity stress directly affects wheat phenological aspects, root growth rate, root/shoot ratio, and total dry matter yield [44]. Therefore, exogenous application of NO can improve salinity tolerance by maintaining the antioxidant and osmolyte metabolism for better growth and yield in salt-exposed seedlings. With this hypothesis, the present investigation was aimed to analyze the possible involvement of exogenous NO in the regulation of growth of wheat through the regulation of physiological, biochemical, and molecular attributes under salt stress.

## 2. Results

### 2.1. Growth Parameters

Results depicting the influence of NaCl stress and NO application on growth parameters such as length and fresh and dry biomass in wheat are shown in (Table 1). Wheat seedlings treated with 100 mM of NaCl showed a significant decline in shoot height and fresh and dry weight, which was, however, mitigated by exogenous application of NO. Relative to control, the observed decline in length and fresh and dry weight of wheat was 36.70%, 38.88% and 41.13% due to 100 mM NaCl. NO application at 50 µM caused an enhancement of 14.25%, 4.82% and 12.98% in length and fresh and dry weight, respectively, while 100 µM NO caused a maximum increase of 22.52%, 24.63% and 40.70% over the control. Application of NO (50 and 100 µM) mitigated the effect of NaCl by 12.26% and 21.59% in shoot length, 16.30% and 32.29% in fresh weight, and 25.09% and 35.89% in dry weight over the NaCl-stressed plants (Table 1).

### 2.2. No Protects Chlorophyll and Photosynthetic Attributes in Salt-Stressed Wheat Plants

**Figure 1:**
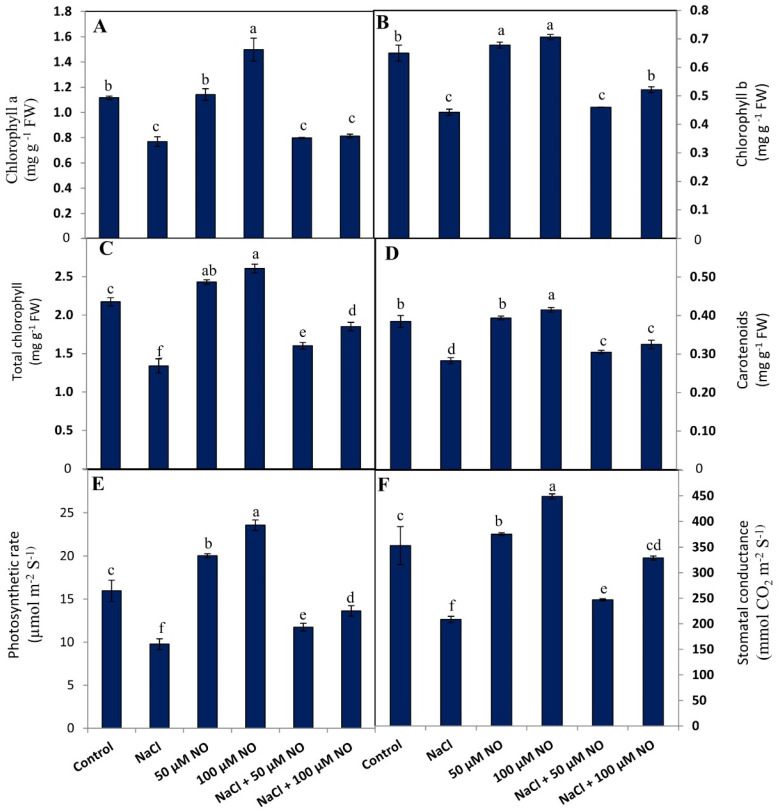
Effect of salinity stress (100 mM NaCl) on (**A**) chlorophyll a, (**B**) chlorophyll b, (**C**) total chlorophyll, (**D**) carotenoids, (**E**) photosynthetic rate, and (**F**) stomatal conductance in wheat (*Triticum aestivum* L.) with and without exogenous application of NO (50 and 100 µM). Data presented are mean (±SE) of three replicates, and bars with different letters denote significant difference at *p* ≤ 0.05.

Salinity stress resulted in a considerable decline in the synthesis of chlorophyll a (Chl a), chlorophyll b (Chl b), carotenoid, and total pigments reflecting in decreased photosynthetic rate and stomatal conductance (Figure 1A–F). Relative to control, NaCl treatment reduced the Chl ‘a’ by 31.1%, Chl ‘b’ by 32%, carotenoids by 26.56%, and total pigments by 38.7%. Application of NO at both concentrations improved the chlorophyll synthesis significantly over the control and also ameliorated the negative effects of salinity by 3.62%, 4.07%, 7.23% and 13.33% with 50 µM NO and by 5.42%, 15.2%, 13.23% and 28.1% with 100 µM NO over the NaCl-treated counterparts. At 100 µM, application of NO (100 mM NaCl + 100 µM NO) ameliorated the negative effect of NaCl on stomatal conductance and photosynthetic efficiency by 28.39% and 36.51%, respectively, over the NaCl-treated plants (Figure 1E,F).

### 2.3. No Maintains Leaf RWC by Improving the Proline and Sugar Content

**Figure 2:**
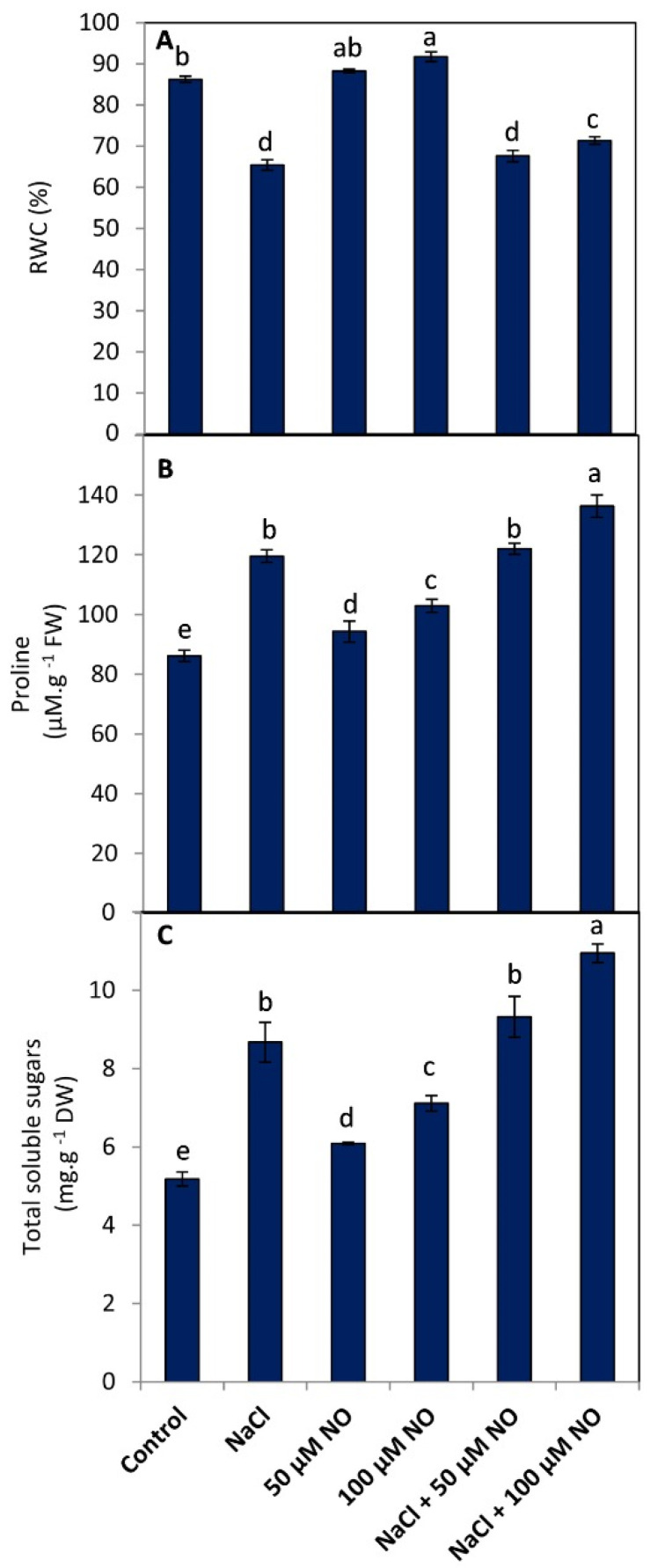
Effect of salinity stress (100 mM NaCl) on (**A**) RWC, (**B**) proline, and (**C**) soluble sugar content in wheat (*Triticum aestivum* L.) with and without exogenous application of NO (50 µM and 100 µM). Data presented are mean (±SE) of three replicates, and bars with different letters denote significant difference at *p* ≤ 0.05.

Results showing the effect of NaCl and NO on the synthesis of proline, sugars, and RWC are depicted in (Figure 2A–C). NO application improved the proline and soluble sugar accumulation in both normal and NaCl-stressed wheat plants conditions, reflecting in increased RWC in them over the controls. RWC was declined in NaCl-stressed plants by 24.12% over control. However, an enhancement of 2.26% and 5.99% was observed with 50 µM and 100 µM NO, respectively. Application of NO to NaCl-stressed plants mitigated the negative effects on RWC by 3.25% at NaCl + 50 µM NO and by 8.27% at NaCl + 100 µM NO over the NaCl (100 mM)-stressed plants. NO-treated plants (100 mM NaCl + 100 µM NO) exhibited maximal accumulation of proline and soluble sugars with an increase of 36.78% and 52.66% over the control plants, resulting in maximal amelioration of salinity-induced decline in RWC (Figure 2A–C).

### 2.4. No Application Reduces Oxidative Damage by Preventing Lipid Peroxidation, Hydrogen Peroxide, and Improving Membrane Stability Index

**Figure 3:**
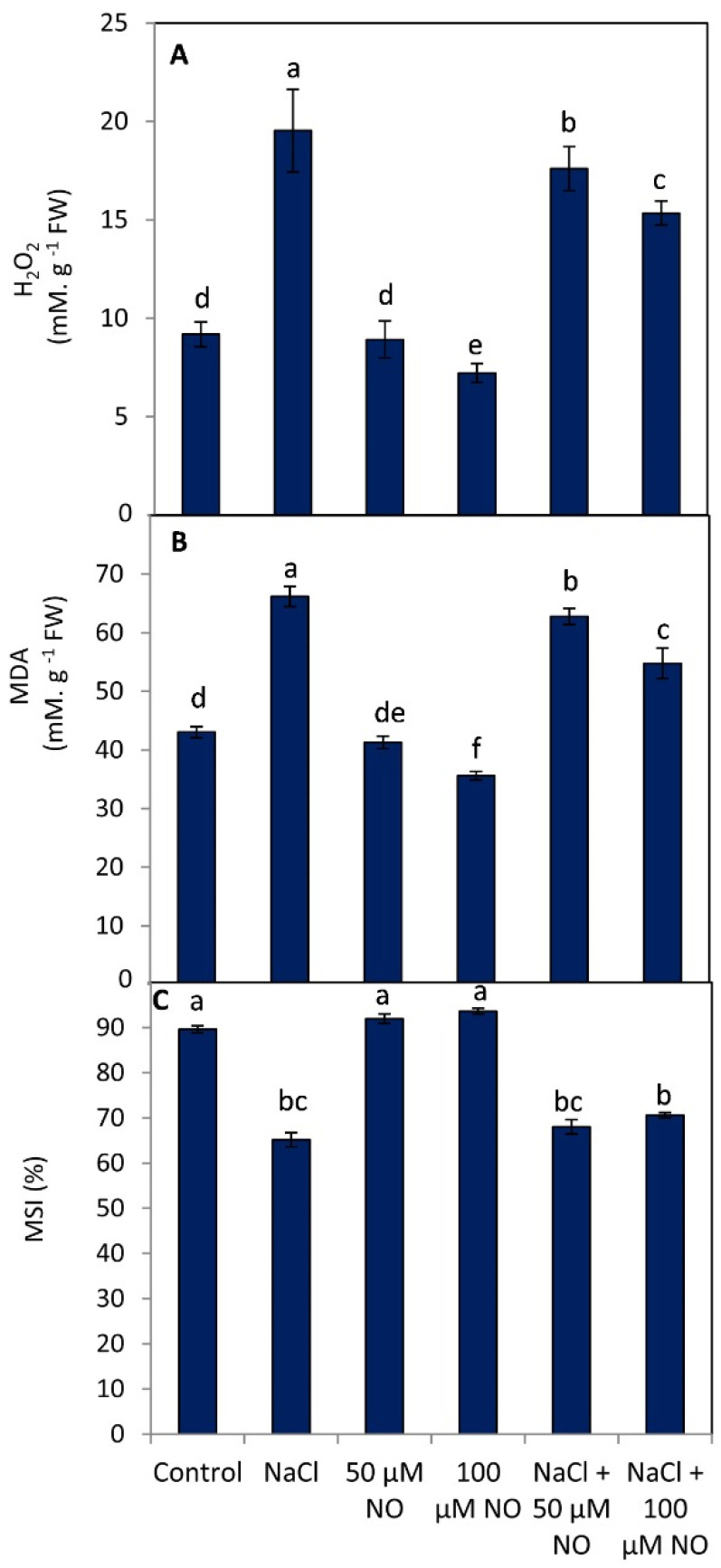
Effect of salinity stress (100 mM NaCl) on (**A**) hydrogen peroxide, (**B**) lipid peroxidation (MDA), and (**C**) membrane stability index (MSI) in wheat (*Triticum aestivum* L.) with and without exogenous application of NO (50 and 100 µM). Data presented are mean (±SE) of three replicates, and bars with different letters denote significant difference at *p* ≤ 0.05.

Exogenous application of NO prevented the oxidative stress by reducing the formation of hydrogen peroxide (H_2_O_2_), and hence the lipid peroxidation resulting in an increased membrane stability index (Figure 3A–C). NaCl stress triggered the generation of H_2_O_2_ by 52.97% over control, causing an increase of 34.97% in lipid peroxidation and subsequently a decline of 27.27% in the membrane stability index in them. Exogenous application of NO at 50 and 100 µM proved significant in declining the NaCl-mediated generation of H_2_O_2_ and hence preventing lipid peroxidation and protecting membrane stability. Maximal protection to wheat seedlings was exhibited by seedlings treated with 100 µM NO, reducing the generation of H_2_O_2_ and lipid peroxidation by 21.35% and 17.24% over the control, and at 100 mM NaCl + 100 µM NO, H_2_O_2_ and lipid peroxidation was ameliorated by 21.14% and 17.19% over the NaCl-stressed plants. The naCl-mediated decline in the membrane stability index was assuaged by 4.2% and 7.62% in 50 µM and 100 µM NO-supplemented seedlings, respectively (Figure 3A–C).

### 2.5. Exogenous NO Up-Regulates Antioxidant System in Salinity-Stressed Wheat

**Figure 4:**
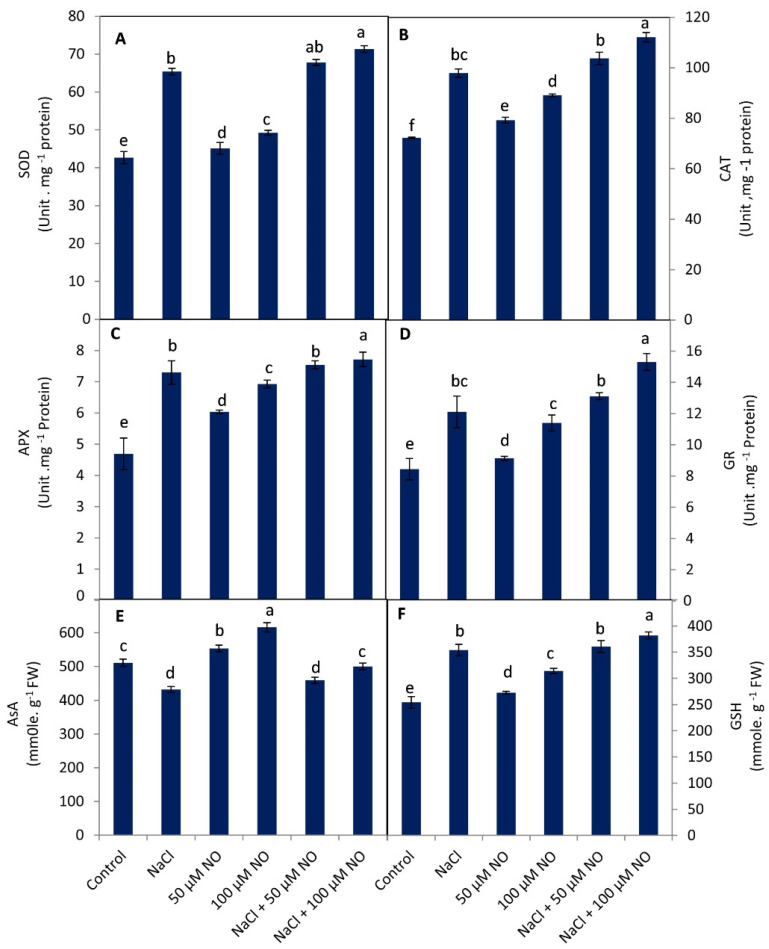
Effect of salinity stress (100 mM NaCl) on activity of (**A**) superoxide dismutase, (**B**) catalase, (**C**) ascorbate peroxidase, (**D**) glutathione reductase, and (**E**) ascorbic acid and (**F**) reduced glutathione content (*Triticum aestivum* L.) with and without exogenous application of NO (50 µM and 100 µM). Data presented are mean (±SE) of three replicates, and bars with different letters denote significant difference at *p* ≤ 0.05.

In both normal and NaCl-exposed wheat seedlings, the activities of the antioxidant enzymes and the contents of non-enzymatic antioxidants studied (glutathione (GSH) and ascorbic acid (ASA)) were observed to increase with the application of NO, and obvious effects were observed with 100 µM NO (Figure 4A–F). Increase in the activities due to 100 mM NaCl was 34.86% in superoxide dismutase (SOD), 26.17% in catalase (CAT), 35.67% ascorbate peroxidase (APX), and 30.3% in glutathione reductase (GR) activity over the control plants. GSH increased by 28.04%, while ASA content decreased by 15.29% due to NaCl treatment. Application of NO increased the activity of SOD, CAT, APX, and GR by 5.54%, 8.83%, 22.22% and 7.45% at 50 µM and by 13.41%, 18.96%, 33.33% and 25.96% at 100 µM over the control plants. Relative to the NaCl-treated seedlings, NaCl + NO-treated ones showed a further increase in the activity of SOD, CAT, APX, and GR, with the increase being more obvious in 100 mM NaCl + 100 µM NO-treated plants showing a percentage increase of 8.27% for SOD, 13.39% for CAT, 5.44% for APX, and 20.9% for GR. ASA was increased by 7.77% and 17.2% by application of 50 µM and 100 µM NO, respectively, and amelioration of 5.88% and 13.6% in ASA content was observed when applied to NaCl-stressed plants. Accumulation of GSH was enhanced maximally in NO-supplemented plants with a percent increase of 29.44% and 33.33% with NaCl + 50 µM NO and NaCl + 100 µM NO, respectively, over the control (Figure 4A–F).

### 2.6. No Improves Uptake of N, K, and Ca under Salinity Stress

Mineral elements were estimated to assess the salt tolerance mediated by exogenous-applied NO. NaCl treatment declined the uptake of nitrogen (N), potassium (K), and calcium (Ca) while increased the accumulation of sodium both in the leaf as well as root (Table 2). Relative to control, N, K, and Ca were observed to decrease by 27.31%, 45.07% and 40.7% in leaf and 25.2%, 32.5% and 34.62% in root tissues, respectively. In leaf tissues, application of NO increased N, K, and Ca by 8.34%, 10.92% and 7.75% at 50 µM and by 19.04%, 25.33% and 28.63% at 100 µM concentrations, respectively. Such a positive influence of NO was also observed when applied to NaCl-treated plants, resulting in significant amelioration of decline in their uptake. Relative to salinity-stressed plants, uptake of N, K, and Ca increased by 23.2%, 33.76% and 32.2%, respectively, with NaCl + 100 µM reflecting in the mitigation of ill effects of salinity stress. In NaCl-treated plants, sodium accumulation increased by 54.06% and 43.58% in leaf and root over the control plants. However, a decline of 7.35% and 31.25% was observed in NO-treated plants at 50 µM and 100 µM, respectively, in leaf tissues, and a similar trend was maintained in root tissues. Application of NO to NaCl-treated plants limited the uptake of Na by 14.35% and 31.12% at NaCl + 50 µM and NaCl + 100 µM, respectively (Table 2).

### 2.7. NO Regulates the Expression of SOS1, NHX1, AQP, OSM-34, and D2-Protein

**Figure 5:**
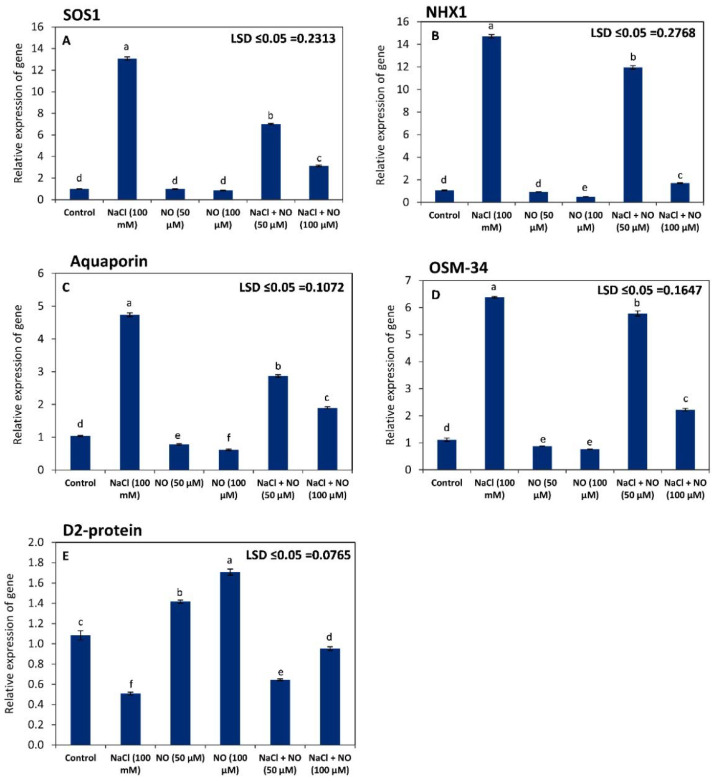
Effect of salinity stress (100 mM NaCl) on relative gene expression of (**A**) SOS1, (**B**) NHX1, (**C**) Aquaporin, (**D**) OSM-34, and (**E**) D2 protein (*Triticum aestivum* L.) with and without exogenous application of NO (50 µM and 100 µM). Data presented are mean (±SE) of three replicates, and bars with different letters denote significant difference at *p* ≤ 0.05.

To further explore the protective effect of exogenous NO on the wheat plants under saline conditions, the relative gene expression of SOS1, NHX1, AQP, OSM-34, and D2 proteins was investigated by real-time (RT)-qPCR (Figure 5). Plants subjected to salt stress (100 mM NaCl) dramatically exhibited an obvious and significant (*p* ≤ 0.05) increase in the relative expression of SOS1, NHX1, AQP, and OSM-34 compared to the non-saline conditions (control). However, an opposite and significant trend was observed with respect to D2-protein.

On the other hand, under salt-stress conditions, NO-treated plants (50 or 100 µM) showed a significant decrease in the relative gene expression of SOS1, NHX1, AQP, and OSM-34 compared to the salt-affected NO-untreated plants (100 mM NaCl), while the relative gene expression of D2-protein revealed an obvious and significant improvement with the treatments of NO at 50 or 100 µM compared to the NO-untreated plants under saline conditions. In this context, the treatment of 100 µM NO was more potent than the other treatment of 50 µM.

## 3. Discussion

Increased salinity levels in soils due to agricultural malpractices have led to the failure of net agricultural productivity and crop survival [45]. Salinity stress has resulted in a considerable decline in crop yields such as soybean [46] and tomato [47]. Hence, the need for the prevention of crop losses to meet the demands of ever-increasing human populations [48]. Accordingly, research investigations are needed to provide some better alternatives for improving the protection mechanisms against stress to enhance the yield. In this connection, we analyzed the efficiency of exogenously supplied NO in enhancing growth through its involvement in the regulation of key physiological and biochemical attributes. Increased salinity concentrations in soil solution potentially reduce the morphological parameters by restricting the cell division, leading to declined cellular elongation and growth [49]. NO has the potential to ameliorate such adverse effects and enhance the stress resilience of plants, which can be ascribed to the key role in stress mitigation [50,51]. Growth parameters showed an increase with increasing concentration of NO from 50 µM to 100 µM and also ameliorated the decline triggered by salinity (100 mM NaCl) to a considerable extent (Table 1). Our results are in corroboration with Fatma and Khan (2014) [41] for mustard, Ahmad et al. (2016) [52] for chickpea, and Fan et al. (2013) [53] for cucumber. The ameliorative impact of exogenous NO on the growth parameters is believed to be due to its cumulative effect on the uptake and accumulation of key mineral nutrients [54]. In the present study, exogenous application of NO prevented excess accumulation of Na in upper plant parts, preventing the intensity of NaCl-mediated generation of oxidative stress in wheat plants (Table 2). Exogenously sourced NO triggers the vacuolar H^+^-ATPase and increased Na^+^/H^+^ antiport activity, thereby reflecting inefficient compartmentalization of Na^+^ [55].

Similar to our observation, the positive implication of NO in improving the K^+^, Ca^2+^, and Mg^2+^ content and preventing salinity-triggered decline has been demonstrated in *Gossypium hirsutum* [56]. Exogenously sourced NO increased uptake of N, K, and Ca, reaching maximal values with 100 µM NO under normal conditions and also maintaining its salinity stress amelioration potential. Earlier, it has been demonstrated that NO improves the uptake of N, P, and K in wheat seedlings subjected to cadmium stress [57], reflecting improved photosynthesis. Exogenously applied NO may have led to the maintenance of cytosolic Na concentrations by improving the expression of Na^+^ transporters and H^+^ pumps [58]. In addition, NO application potentiates the Ca-mediated stress amelioration by improving its uptake and partitioning to cellular compartments, and in the present investigation, NO proved beneficial in improving the Ca and K uptake, causing a significant reduction in Na/K ratio [59,60]. Lowering the Na/K ratio strengthens the cellular metabolism and protects enzyme activity, synthesis of metabolites, and yields performance of crop plants [19,61]. Exogenous NO application restricted the uptake of deleterious sodium ions, resulting in a significant decrease in the Na/K ratio by way of increasing mineral uptake. In the present study, NO-mediated reduction in Na/K ratio contributes to reduced susceptibility of wheat plants by mediating the exclusion of the deleterious ions for the maintenance of the cellular osmotic potential [37,60]. NO application was advantageous in improving the chlorophyll and carotenoid pigments at both the concentrations and imparted a positive impact on the photosynthetic efficiency and stomatal conductance. Earlier reports detect the reduction in chlorophyll contents in NaCl-stressed plants [62,63]. Enhancement of photosynthetic capacity following NO treatment was observed in salt-stressed wheat plants by assisting in the synthesis of other pigments protecting components such as cysteine and reduced glutathione that protect the chlorophyll breakdown as well as up-regulation of the activity of enzymes involved in chlorophyll biosynthesis [64,65]. The optimal presence of NO modulates photosynthetic functioning by improving CO_2_ assimilation, photosynthetic rate, and chlorophyll fluorescence characteristic under normal and stressful conditions [66].

Similar to our finding, exposure to salinity stress triggers the accumulation of osmolytes for better protection of cellular functioning [19,67]. Moreover, NO application leads to the maintenance of relative water content by improving the accumulation of proline and soluble sugars to maintain the water potential below the external solution [68]. Overall physiologically, these osmoprotectants reduce cellular osmotic potential [69], and a reduction in the hydraulic conductivity of the membranes occurs, possibly by decreasing the number of water channels (aquaporins) [70]. NO application may have improved the expression of proteins involved in the biosynthesis of osmolytes, mainly proline, and be associated with a decrease in its catabolizing enzymes [71].

Salinity treatment was observed to increase the generation of free radical H_2_O_2_, causing more significant damage to membrane lipids and loss of membrane stability index [72]. For preventing the oxidative damage that is triggered by ROS, plants intensify the ROS-scavenging mechanisms [73]. In the present study, 100 mM NaCl stress raised the hydrogen peroxide and lipid peroxidation level, and NO supplementation in salt-stressed plants maintained higher activity of an antioxidant system (SOD, CAT, APX, and GR) than untreated plants. These results were in accordance with Elkahoui et al. (2005) [74] in *Catharanthus roseus*, Hernandez et al. (2010) [75] in *Brassica oleracea*, and Carrasco-Ríos and Pinto (2014) [76] in *Zea mays.* Earlier evidence from Lamattina et al. (2003) and Corpas and Barroso (2015) [77,78] showed that NO mediated better growth and significant amelioration of the oxidative damage due to increased stabilization of macromolecules such as lipids, proteins, and nucleic acids. Moreover, exogenously supplied NO controlled the production of antioxidants and accumulation of ROS and directly interacted with lipid alkoxyl and peroxyl radicals, leading to preventing the propagation of radical-mediated lipid oxidation [79]. NO-treated plants significantly up-regulated the antioxidant system in imparted quick elimination of excess accumulated free radicals, resulting in the stability of structural and functional aspects of cellular membranes [52,76]. Greater activity of SOD, CAT, APX, and GR has been correlated with improved stress tolerance, and NO-mediated enhancement in their activities will lead to a further reduction in the intensity of oxidative stress [54].

Under saline conditions, achieving ion homeostasis and conserving water status and photosynthesis are the most important challenges for plant growth and development. Therefore, different plant species develop a wide array of defensive mechanisms that can protect them from the destructive effect of salt stress. In the current study, activation of the signaling pathways of the plasma membrane (*SOS1*) and vacuolar (*NHX1*) Na^+^/H^+^ antiporters were observed by increasing the relative gene expression of the salt-stressed plants compared to the unstressed ones (Figure 5). These two proteins have been found to be responsible for excluding Na^+^ ions from the cytosol to outside plasma membrane or inside vacuole, respectively. These responses enable plants to survive under salt stress by avoiding Na^+^ toxicity on different plant metabolisms and maintain ion homeostasis in the cytoplasmic matrix [80]. Aquaporins (AQPs) are well-known membrane channel proteins that are responsible for water, metal ions, gasses, and small neutral solutes transport during biotic and abiotic stresses [81]. Similarly, osmotin (*OSM-34*) is a cysteine-rich protein synthesized in vacuoles to function as an osmoregulator under low water potential [82]. It can also control the oxidative damage induced by ROS, specifically H_2_O_2_, and isolate Na^+^ in the vacuoles during salt stress [83]. Furthermore, overexpression of *OSM-34* has been found to reduce lipid peroxidation and increase the proline content under different stresses [83]. In this study, the overexpression of aquaporin and *OSM-34* in the salt-affected plants reveals the importance of both proteins in the regulation of osmotic potential and keeping plant–water relations under saline conditions. Conversely, exposing plants to salt stress led to diminishing the relative expression of D2-protein, which is considered one of the core proteins in the photosystem II center reaction. This protein is vulnerable to the oxidative damage and photoinhibition process during stress conditions [84].

Applied NO has been found to counteract the detrimental effects of osmotic stressors, i.e., drought [13] and salinity [85,86]. These effects may be due to enhancing the antioxidant capacity, osmotic potential, nutrient homeostasis, and gas exchange [13,86]. In the present investigation, exogenous NO, particularly at 100 µM, led to a significant downregulation in the relative expression of *SOS1, NHX1, AQP,* and *OSM-34* of the salt-affected plants compared to the salt-stressed NO-untreated plants, while an obvious improvement in the expression of D2- protein was observed by the treatments of NO (50 µM and 100 µM) in the salt-stressed plants compared to the NO-untreated plants. These responses may imply that applied NO can preserve photosynthesis, osmotic potential and minimize Na^+^ toxicity in the salt-stressed plants, as there is no need to activate the ionic homeostasis (*SOS1/NHX1*) or osmotic (*AQP/OSM-34*)-related proteins with enhancing the photosynthetic efficiency (D2-protein) of the NO-treated plants under salt stress conditions.

## 4. Materials and Methods

### 4.1. Experimental Design and Treatment

Seeds of *Triticum aestivum* L. were surface-sterilized using 0.5% sodium hypochlorite for 3 min and were repeatedly washed with distilled water and sown in earthen pots having a diameter of 27 cm filled with peat, compost, and sand (4:1:1). At the time of sowing, pots were supplied with 250 mL of full-strength Hoagland’s solution [87]. After seedling growth for 10 days, pots were divided into two groups, and one set was supplied with modified Hoagland’s solution containing 100 mM NaCl, and another set was given normal Hoagland’s solution every alternate day. NO (in the form of sodium nitroprusside as NO donor) at the concentration of 50 µM and 100 µM (10 mL per pot) was applied foliarly to both normal and NaCl-treated sets, and the control was supplied with an equal amount of distilled water and was also maintained. Seedlings were allowed to grow for another 20 days. After 1 month, plants were analyzed for different parameters such as chlorophyll pigments, photosynthetic functioning, oxidative damage attributes, osmolytes, and antioxidant system. The pot was laid in a complete randomized block design with five replications.

### 4.2. Estimation of Photosynthetic Pigments and Measurement of Stomatal Conductance and Photosynthetic Efficiency

Photosynthetic pigments were quantified in fresh leaves after extracting in dimethyl sulfoxide (DMSO), and the optical density of the supernatant was measured by spectrophotometer at 480, 510, 645, 663 nm against DMSO [88]. Photosynthetic efficiency and stomatal conductance were measured in upper fully expanded leaves at 13:00 by using the infrared gas analyzer (CID-340, Photosynthesis System, Bio-Science, Pullman, WA USA).

### 4.3. Determination of Leaf Water Content, Proline, and Soluble Sugars

Relative water content (RWC) was determined by punching leaf discs from fresh treated and normal plants, and their fresh weights were determined. After that, the same leaf discs were kept in Petri dishes containing distilled water for 1 h to gain turgidity. After recording turgid weight, the leaf discs were oven-dried at 80 °C for 24 h to record the dry weight [89]. Calculations were completed using the following formula:

RWC (%) = Fresh weight − Dry weight/Turgid weight − dry weight × 100(1)

For proline, 0.5 g of leaf sample was extracted in 3% (*w/v*) sulphosalicylic acid and was subjected to centrifugation at 3000× *g* for 20 min. A total of 2 mL of the supernatant was reacted with 2 mL of glacial acetic acid and 2 mL of ninhydrin reagent at boiling temperature for 1 h. Subsequently, the samples were kept on an ice bath, and the content of proline was separated using toluene, and absorbance was read spectrophotometrically at 520 nm [90]. Using the data obtained from the standard curve prepared at known concentrations, linear regression is completed (comparing absorbance vs. proline concentration).

For estimation of sugar content, dry plant sample was extracted in boiling ethanol (80 *v*/*v*) and centrifuged for 20 min at 5000× *g*. The concentration of soluble sugars was measured by reacting the extract with anthrone reagent, and optical density was recorded at 585 nm. A standard curve of glucose was used to determine the soluble sugar content [91].

### 4.4. Measurement of Membrane Stability Index, Lipid Peroxidation, and Hydrogen Peroxide

Membrane stability index (MSI) was determined by chopping 0.1 g fresh leaf tissue in test tubes containing 10 mL distilled water. After that, tubes were boiled at 40 °C for recording the electric conductivity (EC_1_), and same tubes were boiled at 100 °C, and again EC (EC_2_) was recorded [92]. Percent MSI was calculated using the formula:

(MSI) = [1 − (EC_1_/EC_2_)] × 100 (2)

Lipid peroxidation was measured by estimating the formation of malonaldehyde (MDA) content. For lipid peroxidation determination, fresh leaves were extracted in trichloroacetic acid (1%, *w/v*, TCA). After centrifugation at 10,000× *g* for 5 min, 1.0 mL supernatant was mixed with 0.5% thiobarbituric acid, and mixture was boiled at 95 °C for half an hour. After that, tubes were kept on ice bath followed by centrifugation for 5 min at 5000× *g* for clarification, and optical density was read at 532 nm and 600 nm [93]. The MDA concentration was determined by dividing the difference in absorbance (A532–A600) by its molar extinction coefficient (155 mM^−1^ cm^−1^), and results expressed as mmol g^−1^ fresh weight.

The concentration of hydrogen peroxide (H_2_O_2_) was estimated by extracting fresh leaf samples in 0.1% (*w/v*) TCA using pestle mortar. After centrifugation at 12,000× *g* for 15 min, a known volume of the supernatant was mixed with 0.5 mL of 10 mM potassium phosphate buffer (pH 7.0) and 1 M potassium iodide (1 mL). Subsequently, the optical density of the mixture was taken at 390 nm [94], and computation was completed using a standard curve of H_2_O_2_.

### 4.5. Assay of Antioxidant Enzymes

Antioxidant enzymes were extracted by homogenizing 5.0 g fresh leaves in chilled pestle and mortar using 50 mM sodium phosphate buffer (pH 7.0) containing 1% (*w/v*) polyvinyl pyrrolidine. The resulting homogenate was used as the enzyme source after centrifugation at 15,000× *g* for 20 min at 4 °C, and the protein content was determined by following [95].

The activity of superoxide dismutase (SOD, EC 1.15.1.1) was determined by adopting the method of [96]. Briefly, enzyme aliquot was incubated under light and dark to monitor the photoreduction of nitroblue tetrazolium (NBT) at 560 nm, and the activity of SOD was expressed as enzyme unit (EU) mg^−1^ protein. One unit of enzyme activity represents the amount of enzyme required for 50% inhibition of NBT reduction at 560 nm. For assaying activity of catalase (CAT, EC 1.11.1.6), [97]’s method was adopted, and change in optical density was recorded at 240 nm. An extinction coefficient of 36 × 10^3^ mM^−l^ cm^−l^ was used for calculation and expressed as EU mg^−1^ protein. For determination of ascorbic peroxidase (APX) activity, 0.1 mL enzyme was added to 1 mL potassium phosphate buffer (100 mM, pH 7.0), 0.1 mM EDTA, 0.5 mM ascorbate, and 0.1 mM H_2_O_2_. The disappearance of H_2_O_2_ was observed as a change in absorbance at 290 nm [98]. The reaction was initiated by addition of hydrogen peroxide, and oxidation of ascorbate was followed by the decrease in absorbance at 290 nm at 30 s interval for 5 min. One unit of APX activity is defined as the amount of enzyme that oxidizes 1 µM of ascorbate per min at room temperature. For determination of glutathione reductase (GR, EC 1.6.4.2), activity change in absorbance was recorded at 340 nm for 3 min following [99]. For calculation of activity, an extinction coefficient of 6.2 mM^−1^ cm^−1^ was used.

### 4.6. Determination of Ascorbate and Reduced Glutathione

For determination of ascorbic acid, fresh leaf samples were macerated in 5% (*w/v*) TCA, and to the extract, 2% (*w/v*) dinitrophenyl-hydrazine and 10% (*w/v*) thiourea were added. The resultant mixture was kept in a boiling-water bath for 15 min and brought to room temperature followed by centrifugation at 1000× *g* for 10 min. For dissolving the resulting pellet, 80% (*v/v*) H_2_SO_4_ was added, and optical density was taken at 530 nm [100]. A standard curve of ascorbic acid was used for calculation.

Reduced glutathione (GSH) was estimated by homogenizing 500 mg fresh leaf tissue in a phosphate buffer. After centrifugation at 3000× *g* for 15 min, 40 µL of 5, 5-dithiobis-2-nitrobenzoic acid was added to 500 µL supernatant and allowed to stand for 10 min. Absorbance was taken at 412 nm [101]. A standard curve of GSH was used for calculation.

### 4.7. Estimation of Mineral Ions

Estimation of mineral ions, including Na, K, and Ca, was completed using the flame photometer after acid digesting the dried tissue [102]. For determining the nitrogen content in treated and untreated tissues, the method of Subbiah and Asija [103] was adopted.

### 4.8. Gene Expression

Total mRNA was isolated from 0.5 g shoot parts of wheat plant of all treatments after 2 weeks of salinity and NO foliar application using Total RNA extraction kit (Sigma-Aldrich) according to the manufacturer’s protocol. The purified RNA was quantitated spectrophotometrically and analyzed on 1% (*w/v*) agarose gel. Reverse transcription of RNA was performed. The reaction mixture contained 10 as oligo dT primer (10 pml/μL), 2.5 μL 5 × buffer, 2.5 μL MgCl_2_, 2.5 μL 2.5 mM dNTPs, 4 μL oligo (dT), 0.2 μL (5 Unit/μL) reverse transcriptase (Promega, Walldorf, Germany), and 2.5 μL RNA. RT-PCR amplification was performed in a thermal cycler PCR, programmed at 42 °C for 1 h and 72 °C for 20 min. Quantitative real-time PCR for Gene expression analysis used a SYBR^®^ Green-based method. Primers of 5 specific genes and housekeeping gene (reference gene) were used in real-time analysis using (Rotor-Gene, Düsseldorf, Germany). A total reaction volume of 20 µL was used. Reactions included 2 µL of template, 10 µL of SYBR Green Master Mix, 2 µL of reverse primer, 2 µL of forward primer, and sterile distilled water for a total volume of 20 µL. PCR assays were performed using the following conditions: 95 °C for 15 min followed by 40 cycles of 95 °C for 30 s and 58 °C for 30 s. The CT of each sample was used to calculate ΔCT values (target gene CT subtracted from β-Actin and tubulin gene CT). The relative gene expression was determined using the 2^−ΔΔCt^ method [104].

### 4.9. Statistical Analysis

Data are mean (±SE) of three replicates, and for testing significance of data, Duncan’s Multiple Range Test was performed using One-Way ANOVA, and the least significant difference (LSD) was calculated at *p* < 0.05.

## 5. Conclusions

Conclusively, it can be said that salinity reduces the growth of wheat through alterations in the physiological and biochemical parameters studied. NaCl treatment increased the lipid peroxidation inducing membrane dysfunction, hence leading to impeding the uptake of important mineral elements. However, the application of NO effectively lessened the negative impact of salinity on growth and physio-biochemical parameters by improving osmolytes and antioxidant metabolism. NO treatment dispelled the salt-stress-mediated ravage by restricting the excess accumulation of Na^+^ and better scavenging of ROS through the up-regulated antioxidant system (enzymatic and non-enzymatic). NO-mediated osmoregulation in wheat plants directly affected the ROS scavenging and the mineral nutrients uptake in wheat, leading to significantly alleviated NaCl stress. This protective effect of NO extended to the molecular level by affecting the relative gene expression of the ionic homeostasis (*SOS1/NHX1*), osmotic (*AQP/OSM-34*), and photosystem II (D2-protein)-related proteins. We recommend that future works focus on how the responses of wheat genotypes contrast in salt tolerance to NO treatment under both normal and salt-stress conditions (in different stages of wheat ontogenesis).

## Figures and Tables

**Table 1:** Effect of salinity stress (100 mM NaCl) on shoot length (cm) and shoot fresh and dry weight (g) in wheat (*Triticum aestivum* L.) with and without exogenous application of NO. Data presented are mean (±SE) of three replicates and different letters denote significant difference at *p* ≤ 0.05.

	Shoot Length (cm)	Shoot Fresh Weight (gm)	Shoot Dry Weight (gm)
Control	22.26 ± 1.006c	4.573 ± 0.417c	1.206 ± 0.103bc
NaCl (100 mM)	14.09 ± 0.899e	2.725 ± 0.453f	0.7071 ± 0.012e
NO 50 µM	25.96 ± 2.65b	4.805 ± 0.116b	1.386 ± 0.055b
NO 100 µM	28.73 ± 1.08a	6.068 ± 0.073a	2.034 ± 0.06a
NaCl + NO 50 µM	16.06 ± 0.125d	3.256 ± 0.169e	0.944 ± 0.05d
NaCl + NO 100 µM	17.97 ± 0.047d	4.025 ± 0.18d	1.103 ± 0.1c

**Table 2:** Effect of salinity stress (100 mM NaCl) on nitrogen, potassium calcium, sodium, and Na/K ratio in wheat (*Triticum aestivum* L.) with and without exogenous application of NO. Data presented are mean (±SE) of three replicates, and different letters denote significant difference at *p* ≤ 0.05.

	N (mg g^−1)^		Na (mg g^−1)^		K (mg g^−1)^		Ca (mg g^−1^)		Na/K %	
	Leaf	Root	Leaf	Root	Leaf	Root	Leaf	Root	Leaf	Root
Control	35.37 ± 1.05c	17.46 ± 1.02c	7.072 ± 0.51d	9.98 ± 0.87d	45.64 ± 0.74c	26.55 ± 1.44c	6.78 ± 0.502c	4.91 ± 0.54c	0.154	0.375
NaCl (100 mM)	25.71 ± 1.05f	13.06 ± 0.96d	15.39 ± 1.15a	17.69 ± 0.6a	25.07 ± 0.27f	17.92 ± 0.89f	4.02 ± 0.327ef	3.21 ± 0.29ef	0.613	0.987
NO 50 µM	38.59 ± 0.61b	19.3 ± 1.12b	6.55 ± 0.37d	7.66 ± 0.42e	51.24 ± 1.22b	30.02 ± 1.04b	7.35 ± 0.168b	5.56 ± 0.38b	0.127	0.255
NO 100 µM	43.69 ± 1.13a	25.31 ± 0.97a	4.86 ± 0.08e	5.51 ± 0.55f	61.13 ± 1.12a	34.75 ± 1.03a	9.5 ± 0.465a	6.93 ± 0.14a	0.079	0.158
NaCl + NO 50 µM	27.95 ± 0.11e	14.92 ± 0.97d	13.18 ± 0.61b	15.29 ± 1.01b	31.41 ± 0.6e	19.43 ± 0.65e	4.58 ± 0.302e	3.6 ± 0.51e	0.419	0.786
NaCl + NO 100 µM	33.48 ± 0.44cd	16.68 ± 1.08c	10.6 ± 0.53c	12.71 ± 0.54c	37.85 ± 0.9d	24.25 ± 0.58cd	5.93 ± 0.153d	4.56 ± 0.4cd	0.28	0.524

## Data Availability

Not applicable.
