# Correction: Alnusairi et al. Exogenous Nitric Oxide Reinforces Photosynthetic Efficiency, Osmolyte, Mineral Uptake, Antioxidant, Expression of Stress-Responsive Genes and Ameliorates the Effects of Salinity Stress in Wheat. *Plants* 2021, *10*, 1693

**DOI:** 10.3390/plants11050576

**Published:** 2022-02-22

**Authors:** Ghalia S. H. Alnusairi, Yasser S. A. Mazrou, Sameer H. Qari, Amr A. Elkelish, Mona H. Soliman, Mohamed Eweis, Khaled Abdelaal, Gomaa Abd El-Samad, Mohamed F. M. Ibrahim, Nihal ElNahhas

**Affiliations:** 1 Department of Biology, College of Science, Jouf University, Sakaka 72388, Saudi Arabia; gshalnusairi@ju.edu.sa; 2 Business Administration Department, Community College, King Khalid University, Guraiger, Abha 62529, Saudi Arabia; ymazrou@kku.edu.sa; 3 Faculty of Agriculture, Tanta University, Tanta 31512, Egypt; 4 Biology Department, Al-Jumum University College, Umm Al-Qura University, Mecca 21955, Saudi Arabia; shqari@uqu.edu.sa; 5 Botany Department, Faculty of Science, Suez Canal University Ismailia, Ismailia 41522, Egypt; amr.elkelish@science.suez.edu.eg; 6 Botany and Microbiology Department, Faculty of Science, Cairo University, Giza 12613, Egypt; amradel807080@googelmail.com; 7 Plant Pathology and Biotechnology Laboratory, Excellence Center (EPCRS), Faculty of Agriculture, Kafrelsheikh University, Kafr Elsheikh 33516, Egypt; khaled.elhaies@gmail.com; 8 Department of Agronomy, Faculty of Agriculture, Ain Shams University, Cairo 11566, Egypt; Gomaa_abdelsamad@agr.asu.edu.eg; 9 Department of Agricultural Botany, Faculty of Agriculture, Ain Shams University, Cairo 11566, Egypt; Ibrahim_mfm@agr.asu.edu.eg; 10 Department of Botany and Microbiology, Faculty of Science, Alexandria University, Alexandria 21526, Egypt; nihal.elnahhas@alexu.edu.eg

In the original publication [1], the acknowledgements section was not included. The acknowledgements are hereby published as follows:
